# Seasonal variation of food security among the Batwa of Kanungu, Uganda

**DOI:** 10.1017/S1368980016002494

**Published:** 2016-09-13

**Authors:** Kaitlin Patterson, Lea Berrang-Ford, Shuaib Lwasa, Didacus B Namanya, James Ford, Fortunate Twebaze, Sierra Clark, Blánaid Donnelly, Sherilee L Harper

**Affiliations:** 1 Department of Population Medicine, Ontario Veterinary College, University of Guelph, Guelph, Ontario, Canada, N1G 2W1; 2 Department of Geography, McGill University, Montreal, Quebec, Canada; 3 Indigenous Health Adaptation to Climate Change Research Team †; 4 Department of Geography, Makerere University, Kampala, Uganda; 5 Ministry of Health, Kampala, Uganda

**Keywords:** Seasonal variation, Food security, Indigenous populations, Social determinants of health, Mixed methods

## Abstract

**Objective:**

Climate change is projected to increase the burden of food insecurity (FI) globally, particularly among populations that depend on subsistence agriculture. The impacts of climate change will have disproportionate effects on populations with higher existing vulnerability. Indigenous people consistently experience higher levels of FI than their non-Indigenous counterparts and are more likely to be dependent upon land-based resources. The present study aimed to understand the sensitivity of the food system of an Indigenous African population, the Batwa of Kanungu District, Uganda, to seasonal variation.

**Design:**

A concurrent, mixed methods (quantitative and qualitative) design was used. Six cross-sectional retrospective surveys, conducted between January 2013 and April 2014, provided quantitative data to examine the seasonal variation of self-reported household FI. This was complemented by qualitative data from focus group discussions and semi-structured interviews collected between June and August 2014.

**Setting:**

Ten rural Indigenous communities in Kanungu District, Uganda.

**Subjects:**

FI data were collected from 130 Indigenous Batwa Pygmy households. Qualitative methods involved Batwa community members, local key informants, health workers and governmental representatives.

**Results:**

The dry season was associated with increased FI among the Batwa in the quantitative surveys and in the qualitative interviews. During the dry season, the majority of Batwa households reported greater difficulty in acquiring sufficient quantities and quality of food. However, the qualitative data indicated that the effect of seasonal variation on FI was modified by employment, wealth and community location.

**Conclusions:**

These findings highlight the role social factors play in mediating seasonal impacts on FI and support calls to treat climate associations with health outcomes as non-stationary and mediated by social sensitivity.

Populations already experiencing high food insecurity (FI), particularly those engaged in subsistence agriculture, have been identified as among the most vulnerable to the health impacts of climate change^(^ ^1^ ^,^ ^2^ ^)^. Predictions suggest that sub-Saharan Africa will be particularly affected by climatic events such as extreme drought, increased temperatures and unpredictable precipitation, with implications for agricultural productivity, food and water scarcity, and FI^(^ ^3^ ^–^ ^5^ ^)^. Climate change impacts will be mediated by existing socio-economic variation within populations^(^ ^6^ ^,^ ^7^ ^)^, including social gradients (poverty, inequality, barriers to access), economic conditions (price or demand increases, food shortages) and conflict (interrupted supply routes, decreased safety)^(^ ^8^ ^,^ ^9^ ^)^.

FI arises when there is sufficient stress on food systems such that households’ access to and quality or quantity of food resources are impeded^(^ ^10^ ^)^. FI in itself is a negative outcome and it can also be a distal determinant of other negative health outcomes. Populations who are food insecure often have higher rates of malnutrition, stunting and wasting, mental stress, greater risk of infection and higher rates of chronic illness^(^ ^11^ ^,^ ^12^ ^)^. Increases in FI can thus lead to further deterioration of health among vulnerable populations through indirect effects and positive feedback mechanisms^(^ ^13^ ^,^ ^14^ ^)^. To this end, the Fifth Assessment Report of the Intergovernmental Panel on Climate Change stated (with ‘high confidence’) that ‘the interaction of climate change with food security can exacerbate malnutrition, increasing vulnerability of individuals to a range of diseases’ (p. 1024)^(^ ^4^ ^)^.

Sub-Saharan Africa has been identified as one of the most vulnerable regions to the impacts of climate change on FI^(^ ^4^ ^,^ ^15^ ^,^ ^16^ ^)^. In Uganda, adverse health effects of climate change are already being observed, including increased FI and malnutrition^(^ ^17^ ^–^ ^19^ ^)^. The highest rates of chronic FI in the published literature have been recorded among the Batwa of Kanungu District, Uganda, a highly impoverished Indigenous population where 97 % of households were found to be severely food insecure^(^ ^20^ ^)^. These results are supported by literature highlighting the high vulnerability and sensitivity of Batwa health and consistently poorer health outcomes compared with their non-Indigenous neighbours (Bakiga)^(^ ^18^ ^,^ ^21^ ^–^ ^23^ ^)^. The Batwa are an Indigenous Pygmy population that resides throughout central Africa. Traditionally, the Batwa were hunter-gatherers; their nomadic lifestyle facilitated relocation when food sources were scarce, and reduced issues of sanitation and resource depletion^(^ ^23^ ^)^. Following the establishment of Bwindi Impenetrable National Park in 1991, the Batwa were forcibly evicted from their forest homes and were forced to transition into agrarian livelihoods^(^ ^24^ ^)^. Most Batwa were not compensated for this forced relocation, they lacked any historic experience or expertise in agriculture, and had limited exposure to a cash economy^(^ ^25^ ^)^. The bulk of the displaced Batwa in Kanungu District currently live in settlements or land trusts donated and supported by non-governmental organizations and private donors, particularly the Batwa Development Programme.

The main food source for the Batwa during the rainy season is subsistence agriculture: crop cultivation and small livestock rearing. During the dry season household agriculture is supported by food bartered in exchange for manual labour, trade with other farms, or cash earned from employment used to purchase food at the market. Some Batwa engage in low-paying manual labour, working as porters, cleaners, cooks, diggers, tea collectors, brick makers and cultural dancers, or selling handcrafts to tourists. Namara^(^ ^26^ ^)^ presents the most recent estimate of Batwa income ($US 97 annual per capita income or $US 0·26 per day), which is substantially lower than the overall Ugandan per capita income ($US 362 per annum or $US 0·99 per day). Substantial inequities with regard to access to education are also evident; in 2012 the adult literacy rate for the Batwa living in Kanungu District was <12 %^(^ ^27^ ^)^ compared with >75 % among the neighbouring non-Indigenous populations in the Southwestern Province^(^ ^18^ ^,^ ^28^ ^)^. These factors, combined with persistent ethnic discrimination and relatively unsuccessful adjustment to agricultural livelihoods and the cash economy, contribute to the Batwa displaying some of the lowest health indicators in the country; they have been highlighted as one of the world’s most vulnerable populations^(^ ^29^ ^)^.

The severity of FI among the Batwa of Kanungu District has been reported, with 97 % of households found to be food insecure and 84 % showing very low food security (the most severe category of the US Department of Agriculture’s (USDA) Household Food Security Survey Module (HFSSM))^(^ ^18^ ^)^. The level of FI found among the Batwa is substantially higher than the national Ugandan average of 20 %^(^ ^30^ ^)^ and the highest that has so far been published in the peer-reviewed literature. Batwa agricultural practices, like in much of Uganda, are associated with the seasonal wet and dry months that dictate planting and harvesting. Predictions of climate change in Uganda include increased and unpredictable rainfall, rising temperatures and more frequent extreme-weather events^(^ ^17^ ^,^ ^31^ ^)^. Little is known about how climate change will manifest locally due to a lack of meteorological and social monitoring and data collection; however, regional models and community-based research indicate that Kanungu District will face rising temperatures, an increase in extreme weather and a change in precipitation^(^ ^18^ ^,^ ^32^ ^,^ ^33^ ^)^.

There is negligible place-based research evaluating the extent to which climate change will affect FI among vulnerable Indigenous populations in Africa^(^ ^16^ ^,^ ^18^ ^)^. Projections of future impacts on FI and planning for pathways to adaptation are predicated on an understanding of how current food systems are affected by environmental change and seasonal variation^(^ ^34^ ^,^ ^35^ ^)^. This understanding includes estimates of seasonal effect, but also a comprehension of the causal mechanisms by which the impacts of seasonal signals manifest through – and are mediated by – social determinants of health. We contribute to this research gap by critically assessing and characterizing seasonal variation in Batwa food systems. Objectives include: (i) to assess the impact of seasonal signals on FI; (ii) to characterize the lived experience and perceptions of seasonal variation of FI; and (iii) to analyse potential associations mediating factors between season and food.

## Methods

### Study location

**Fig. 1:**
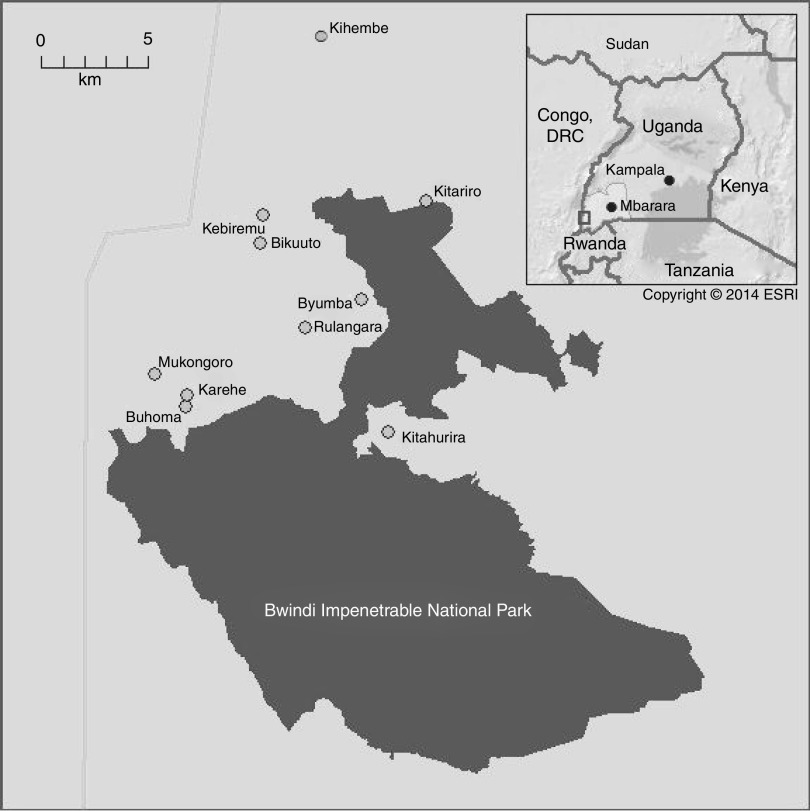
Map of Batwa communities in Kanungu District, Uganda

This research was situated in the District of Kanungu in Southwestern Uganda (Fig. 1). As of 2013, there were approximately 750 Batwa living in ten communities in Kanungu District (assessed during a pilot study in 2010). These communities were participating as partners in an ongoing research project, the ‘Indigenous Health Adaptation to Climate Change’ project (IHACC; www.ihacc.ca), with parallel sites in the Peruvian Amazon (Shawi, Shipibo) and the Canadian Arctic (Inuit).

### Research approach

A concurrent mixed-methods design was used, which involved collecting and analysing both quantitative and qualitative data, and then combining the results for triangulation, complementarity and expansion to better understand the lived experience of FI among Batwa communities^(^ ^36^ ^)^. Herein we employ an interpretativst paradigm which acknowledges that there is no ‘comprehensive truth’ and permits an understanding of multiple ‘experiences’ and ‘perceptions’^(^ ^37^ ^,^ ^38^ ^)^. The quantitative data provided a population-level perspective of FI, while the qualitative data provided an in-depth understanding of the lived experience of FI in Batwa communities.

Climate or seasonal variation is largely responsible for intra-annual variation in agricultural yields. Assessing non-climatic determinants of health is a key component in analysing a population’s vulnerability^(^ ^39^ ^)^. Populations that are poor or that have high health burdens are very sensitive to external stressors like climatic impacts and tend to have lower adaptive capacity^(^ ^4^ ^,^ ^39^ ^)^. Sensitivity to these seasonal signals, though imperfect, represents a proxy for sensitivity to long-term climate change. Such proxies provide a lens through which the role of climatic factors in affecting food systems can be characterized, providing a basis for understanding the potential implications of future change. This approach is referred to in the literature as a ‘temporal analogue’ and is frequently used in climate change vulnerability research^(^ ^34^ ^,^ ^35^ ^,^ ^40^ ^)^. Here, we used a longitudinal study design to provide multiple measures from each season, as shorter-term studies can be skewed by conditions during the study period and may not demonstrate longer-term trends^(^ ^13^ ^)^.

### Quantitative data collection

To quantitatively investigate potential seasonal signals and their impact on FI, six retrospective cross-sectional surveys from all ten Batwa communities in Kanungu District (767 questionnaires) were analysed. Three surveys occurred in the dry season (January 2013, July 2013 and January 2014) and three during the rainy season (April 2013, November 2013 and April 2014). Due to the small size of the Batwa population, an open-cohort census of all Batwa households in the district was attempted. The household response rate varied between 95 and 99 % over the six survey administrations. Three questionnaire instruments were used to collect: (i) individual characteristics and predictors; (ii) household characteristics and predictors; and (iii) household FI status. The individual questionnaire was administered to all members of the Batwa community. The household questionnaire was administered to those who self-identified as the household head, or, in their absence, their spouse or eldest child >18 years. The FI questionnaire was administered to those who self-identified as the head of household food preparation; if they were unavailable, other suitable household members familiar with household food preparation were selected. Questionnaires were conducted orally in Rukiga, the local language, with responses recorded on a paper questionnaire. Community-level predictors were collected in consultation with partners and key informants, and were used to examine the community-level effects that impact FI, including: crop raiding, land quality, market access and landscape type.

#### Dependent variable

The HFSSM, developed by the USDA, was selected to measure FI as it is has been validated for use among vulnerable and Indigenous populations^(^ ^41^ ^)^. We used a 3-month recall period and employed both the standard USDA scoring system (to allow comparison with international research employing this method) and an alternative Adapted Vulnerability Population Score (AVPS) developed by Patterson^(^ ^20^ ^)^ for this population (to capture greater variation in FI) in our analyses of seasonal variation. While the USDA scoring system provides four categorical outcomes for FI (i.e. high, marginal, low and very low food security), the AVPS uses a 26-point scale to capture variation along a continuous gradient.

#### Analysis

Descriptive statistics were used to examine the severity of FI and longitudinal patterns. To investigate the seasonal variation of specific components of FI among the Batwa, univariate logistic regression models examined the unconditional relationship between seasonal variation and each of the ten (eighteen for households with children) questions in the HFSSM. Other variables related to FI such as employment were assessed for seasonal variation using univariate testing. To assess the seasonal variation of FI, we constructed a multilevel linear regression model with a backwards stepwise approach, outlined in Patterson^(^ ^20^ ^)^, using the AVPS continuous variable as the FI outcome variable and accounting for significant household- and community-level predictors of FI. Identification of key variables was guided by Patterson^(^ ^20^ ^)^ and included: wealth, female education, presence of chronic disease, number of dependents, crop raiding and access to markets. The linear model was fitted using random intercepts to account for (i) repeated measures of households across seasons (170 households) and (ii) clustering at the community level (ten communities). The best-fit model was determined by the Akaike information criterion. Post-estimation indicated that the model fit the data appropriately using Pearson’s residuals assumptions of normality and homogeneity of variance for the best linear unbiased predictors. All data analyses were conducted in the statistical software package Stata version 13.

### Qualitative data collection and analysis

Fourteen focus group discussions (FGD) were conducted in seven communities from June to August 2014 (dry season). A discussion guide was developed and reviewed by local research partners and key informants, and then piloted in one community. Question wording was chosen specifically to ensure comprehension in Rukiga (the language of the FGD participants) and to ensure cultural appropriateness. The guide consisted of open-ended questions capturing data on the lived experience of FI as well as perceptions of food security and seasonality. Community chairpersons were approached before the interviewing day to seek permission. On the FGD day, participants were purposively invited to reflect a range of FI levels based on their responses to the household FI questionnaire. However, any additional adult community members were welcomed to participate if they self-selected to join. Groups ranged between three and ten individuals, depending on community size and interest to participate. FGD ranged from 31 to 66 min and lasted an average of 45 min, for a total of 637 interview-minutes.

In addition, fifteen interviews with representatives from health, government, non-government organizations, religious and community sectors were carried out. Semi-structured interviews provided a framework for the interview, but were flexible enough to accommodate the variation in expertise, allowing for elaboration or omission of questions^(^ ^42^ ^)^. The key informant interview guide covered Batwa health and food systems, FI, seasonal variation and the possible implications of climate change. The interviews ranged from 17 to 38 min, lasting an average of 25 min, for a total of 329 interview-minutes. All key informants were asked to choose their preferred language for the interview (English or Rukiga) and all chose to be interviewed in English.

The positionality of the researcher and research team was acknowledged reflexively throughout the research as it can actively change the data collected and the way they are subsequently interpreted^(^ ^43^ ^)^. FGD were used within the Indigenous communities to minimize imbalances in power; groups were further divided by gender to allow for a dynamic where Indigenous women and men were the majority and in control of the conversation. Group discussions were facilitated by Fortunate Twebaze (proficient in both Rukiga and English) and occurred within each community at a communal gathering place. Memoing was utilized to highlight main themes, colloquialisms and tone, and member checking to ensure transcripts reflected participants’ thoughts and ideas accurately and appropriately^(^ ^43^ ^)^. All semi-structured interviews and FGD were audio recorded with permission. The FGD conducted in Rukiga were orally translated and transcribed, using techniques outlined by Lopez *et al.* ^(^ ^45^ ^)^ for recording and assessing data in a second language. Coding and memoing were used to analyse the interview data; these approaches have been validated as appropriate and methodologically rigorous to extract meaning from text^(^ ^44^ ^)^. Following the principles outlined by Fereday and Muir-Cochrane^(^ ^45^ ^)^, both deductive and inductive coding were used to classify and categorize data from the FGD and key informants using the software Atlas.ti version 6.2. The coding supported the analysis of the qualitative data to identify causal mechanisms linking seasonal variation and FI.

## Results

### The Batwa are chronically food insecure

**Fig. 2:**
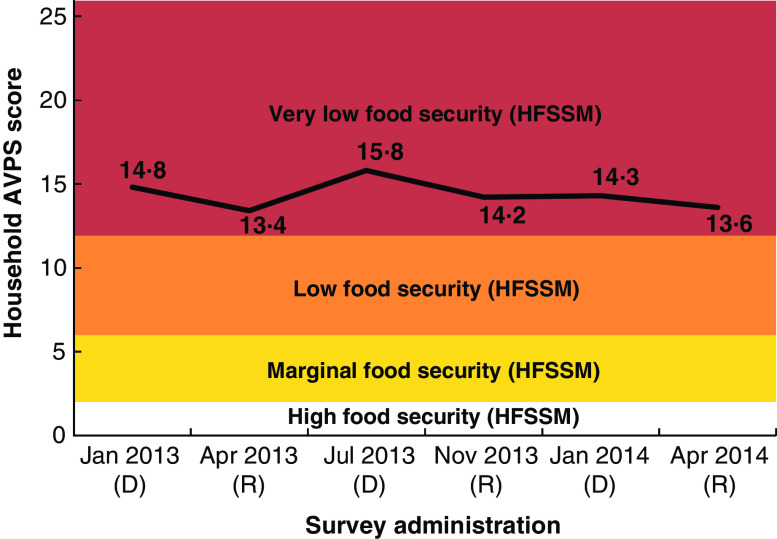
(colour online) Mean Adapted Vulnerable Populations Score (AVPS) by season (D, dry; R, rainy), compared with Household Food Security Survey Module (HFSSM) categorizations, in Batwa Pygmy households (*n* 130) from ten rural Indigenous communities in Kanungu District, Uganda, January 2013–April 2014. Figure demonstrates why variation between seasons was not detectable using the HFSSM: variation occurred only within the most severe category of food insecurity. The black line denotes the mean AVPS across surveys

The prevalence and severity of FI was high across all surveys (Fig. 2). The proportion of households with a USDA rating of ‘very high FI’ ranged from 79 to 92 %. Similarly, the mean AVPS ranged from 13·4 to 15·8, again among the ranges associated with high levels of FI (Table 1). This finding was supported by reports from FGD of chronic FI: ‘These days we are badly off in terms of food. You are about to hear of some of us dying because we don’t have enough food.’ One participant described the effects of both acute and chronic FI observed among children: ‘… when children are not eating well they become more red [malnourished]. You find when their cheeks are swollen and their hair turns brown [lightens], all that is because they are not eating well.’

**Table 1:** Severity of Adapted Vulnerable Populations Score (AVPS) by survey administration among Batwa Pygmy households (*n* 130) from ten rural Indigenous communities in Kanungu District, Uganda, during six surveys

	Jan 2013 (D)	Apr 2013 (R)	Jul 2013 (D)	Nov 2013 (R)	Jan 2014 (D)	Apr 2014 (R)	Dry (mean of Jan/Jul 2013/Jan 2014)	Rainy (mean Apr/Nov 2013/Apr 2014)
Total responses	130	125	131	124	131	130	388	379
Mean AVPS*	14·8	13·4	15·8	14·2	14·3	13·6	15·0	13·8
sd	4·7	4·5	6·2	6·1	6·2	6·0	5·8	5·6
Range of AVPS*	0–25	1–22	1–26	0–25	0–23	0–25	0–26	0–25

R, rainy season; D, dry season.

* As per Patterson^(^ ^20^ ^)^.

The longitudinal surveys indicated that Batwa FI reduced modestly over the study period between January 2013 and April 2014 (*P*<0·05; Fig. 2). However, FGD participants did not perceive any reductions in FI over this time period (January 2013–April 2014). Despite this, some participants reported optimism about the future as a result of improving access to education for their children and increased experience with agrarian practices within their communities. One FGD participant explained: ‘Today we’ve learned digging and growing crops to get food compared to when we were in the forest … This tells that in future and after adopting farming we may not lack food for our families. Because we are trying to adapt to the new life.’

### Food insecurity is most severe during the dry season

FI among households was higher in the dry seasons compared with the rainy and harvest seasons (see online supplementary material, Supplemental Table 1). Even during the harvest season (May/June, November/December), however, FI was severe. The HFSSM categories were not able to detect seasonal shifts in the severity of FI, given that the majority of households were categorized in the highest FI category across all seasons. When using the AVPS, the highest mean scores (indicating higher FI) were consistently recorded during the dry season and the lowest mean scores (indicating lower FI) were recorded during the rainy seasons (Table 1). Focus groups and key informants also reported that FI was higher during the dry season compared with the rainy season. One participant stated: ‘We are really badly off this [dry] season and we don’t have enough to eat.’ A former nurse from the Bwindi Community Hospital noted, for example, that malnutrition cases were higher in the dry season: ‘… during the harvest seasons where most of the people have harvested, there are low cases of malnutrition, but during the sunny seasons there are high cases of malnutrition.’ Consistent with these reports, the multivariable model found that AVPS-measured FI increased significantly during the dry season by 1·13 points (95 % CI 0·4, 1·9) on a scale of 26, or 4 %. Although the magnitude of the difference was relatively small (Fig. 2, Supplemental Table 1), the model indicated that our binary measure of seasonal variation had a stronger effect than other variables with high theorized importance for FI in the published literature, including adult female education, presence of chronic disease and access to markets (Supplemental Table 1).

**Fig. 3:**
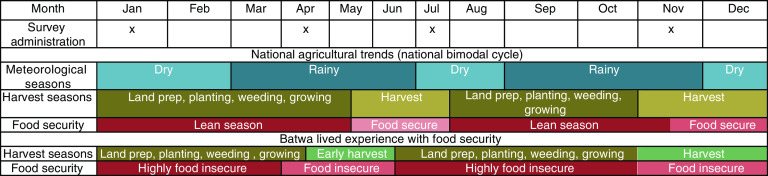
(colour online) Comparison of Southwestern Ugandan seasons and harvesting cycles. Surveys were administered between January 2013 and April 2014 (Jan 2013, Apr 2013, Jul 2013, Nov 2013, Jan 2014 and Apr 2014). National data were extracted from the Global Information and Early Warning System (2015)^(^ ^70^ ^)^ and the Famine Early Warning System Network (2014)^(^ ^71^ ^)^. Batwa data were collected from key informants and focus group discussions, and validated by cross-referencing with local data from the Uganda Wildlife Authority for Buhoma and Rushama stations

Focus groups and key informants reported that these seasonal differences were primarily due to agricultural cycles revolving around land preparation, planting, growing and harvesting (Fig. 3). These cycles were dictated by the rainy and dry seasons; changes in timing, length and intensity of either the rainy or dry months impact harvest yields. The study period reflected typical seasons and was meteorologically comparable to the regional average; there were no extreme atypical events that impacted the region or communities over the study period. Focus groups and key informants stated that digging (agricultural labour for Bakiga) or growing crops in small gardens were the primary strategies employed by households in most communities to access food. During the harvest season, FGD respondents stated that there was greater food availability, lower prices and increased variety of food. Participants highlighted that a lack of food, particularly during the planting season, at the household, community and regional levels, caused prices to increase in the local market. One FGD participant described the pre-harvest experience: ‘Even in markets there is no food and the little that is there is expensive.’ A coping strategy identified to manage the increases in prices and lack of supply in markets was to harvest household crops early. As one participant explained, ‘[B]ecause of hunger we harvest food crops even when they have not matured. When they get finished we go and work for the Bakiga to get food crops. Bakiga always have many fields where we can work to get food and they grow a lot of food crops.’ The AVPS-measured FI captures this strategy: FI begins to improve pre-harvest due to early collection of yields and continues to increase as regional harvesting begins, improving supply and lowering prices in local market.

Among specific components of the FI score, some were more impacted by seasonal signals than others (see online supplementary material, Supplemental Table 2). The majority of households reported hunger year-round; however, households were more likely to report adult hunger during the dry season than during the rainy season. Notably, the seasonal variation of food availability reported by communities and key informants during qualitative data collection was not significant in the quantitative logistic models (Supplemental Table 2): the number of households reporting concern or experiences with running out of food before they could acquire more was not significantly different between the rainy and dry seasons. FGD participants discussed having less variety in diet during the dry season, but that availability and quantity of food were chronically low. Beans, *posho* (boiled maize flour), *matoke* (mashed plantain) and potatoes comprised the majority of energy intake year-round, with very limited food diversity at baseline. This lack of seasonal impact documented in the surveys was supported by key informants, noting that Batwa were rarely able to secure meat and mostly subsisted on *posho* and beans year-round. Further illustrating this pattern, a Batwa participant noted, ‘Meat is hard to get and it is too expensive. I’ve spent about a year without eating meat’ (FGD).

Consistent with findings by Patterson^(^ ^20^ ^)^ that children are protected from FI by adults in the household, we found evidence that this protection also applied to seasonal variation in FI (see online supplementary material, Supplemental Table 2). In contrast to significant seasonal variation in some of the general adult FI questions, only one of the HFSSM items concerned with children varied significantly by season: households reported they were less likely to feed children lower-quality foods during the dry season. However, this finding was not reflected in the FGD. While FI was high for both adults and children during all seasons, the burden due to seasonal variation was predominantly borne by household adults. A focus group participant highlighted this protective strategy: ‘What I do is, mak[e] sure my children get food and we can even eat it all and don’t keep anything for my husband.’ Reports of re-allocation of food within a household were common: ‘The little food we get we try to give it to the children’ (FGD participant).

### Agricultural yields are constrained by severe weather events and socio-economic barriers

Focus group participants and key informants, while optimistic about agriculture, extensively discussed both socio-economic and environmental constraints to agricultural yields. Land size and fertility were often brought up as constraints on agricultural yields and contributors to FI. Focus groups and key informants observed poorer fertility of Batwa plots compared with the regional standard. The FGD and key informants further stated that households were practising ‘... over-cultivation, that depreciates our land … [and] doesn’t yield well ...’ Damaging practices and over-cultivation were often because ‘... [the Batwa] don’t have enough land.’

Focus groups and key informants reported that the increases in availability, access and quality of food during the harvest season were highly dependent on the type of crops being grown and on the absence of extreme weather events (e.g. drought, hail), pests and crop raiding. Extreme weather events in both the dry and rainy seasons were the most frequently mentioned hazards for FI. ‘During the dry season, many food crops dry up and then you can’t have much to eat. It varies because in the last season when we had planted millet it was raining heavily and all the seeds were swept off by running water’ (FGD). Droughts were perceived to be particularly difficult as they impact both food and water security: ‘We are affected by drought [a month or longer], like once a year. Dry seasons don’t only affect the crops but also our water sources dry up, yet most of the work and activities we do at home all rely on using water’ (FGD).

Awareness of potential coping strategies was common; crop rotation (including fallow years), crop diversity, cash cropping, animal husbandry, agricultural inputs associated with improved yields, saving during the harvest season and long-term planning were all identified as potential strategies in FGD. However, lack of land was said to restrict implementation of these coping mechanisms. For example, different harvesting cycles of vegetables and legumes can provide food year-round if timed appropriately, but small plots cannot support such a diversity of crops. During one FGD, participants stated they were unable to produce adequate crop yields, ‘[w]e grow food crops and after harvesting we survive on them for about a month and they get finished. We can never grow crops that can last for over a year.’ The FGD participants and key informants remarked that the non-Indigenous neighbouring (Bakiga) population were able to plant both staple and cash crops (coffee, tea), which led to food security and improved cash wealth. FGD participants stated, ‘[w]e want to grow all types just like the Bakiga.’ In another FGD a participant said, ‘[i]n fact, I wish we could be given more land and grow cash crops like tea so we can get income.’

Finally, the Batwa are a unique group in the region as they lack traditional knowledge regarding agriculture. A key informant from Bwindi Community Hospital viewed their lack of knowledge and experience as a cause of FI: ‘Even if they had that land, they don’t have that knowledge to cultivate their own food and be self-reliable and self-dependent on that food. Whereas … the non-Batwa they have the knowledge … and the land.’ Since their eviction from the forest, the Batwa have been forced to adapt to agricultural livelihoods, with constraints accessing tools and seeds, and a lack of community knowledge regarding agricultural cycles, food/cash crops and harvesting. A key intervention to reduce FI identified by both the focus group participants and key informants was agricultural training. An FGD participant stated that they wanted ‘… a project that can help [us] and look after [us] by sending people to educate [us] on better ways of farming.’

### Socio-economic factors mediate seasonal signals on food insecurity

Socio-economic factors and their interaction with seasonal variation mediated FI among the Batwa of Kanungu District. Employment opportunities were significantly higher during the dry season; households were 1·65 times more likely to report having employed household members during the dry season, but employment was not found to be significant in any of the quantitative FI models. ‘Digging’ (manual agricultural labour) was identified as the primary source of employment but cannot occur when it is raining: ‘That kind of rain also affects our work because it starts in the morning when we want to go and work for food. And once it has started raining you can’t move to any field’ (FGD). Other income-generating activities that the Batwa engage in such as collecting firewood, brick making and collecting tea are all also weather dependent.

In some communities located in close proximity to tourist sites, key informants and focus group participants stated that some households preferred alternative sources of income to acquire food rather than participating in agriculture: ‘… some people don’t mind [bother] about growing crops’ (FGD). Frustration with lack of yields and long hours for small remuneration were key reasons households sought alternative sources of income. A focus group participant highlighted the frustrations associated with low yields: ‘[w]e get discouraged that we grow crops and they don’t yield … even you would get discouraged. If nothing grew, you would give up too.’ Focus groups and key informants mentioned tourism was an attractive alternative to agriculture as returns are higher and immediate compared with growing crops and ‘digging’: ‘Crop growing is … practised but it doesn’t help us so fast, like handcrafts. Even if we grow crops when we are not practising handcrafts we can’t have anything to eat until the crops grow’ (FGD). Compared with the 4000 UGX (Ugandan Shillings; ~$US 1·30) average for a day of intensive ‘digging’, dancing for an hour can generate 10 000 UGX (~$US 3·33) or more, and crafts can sell for 5000–50 000 UGX (~$US 1·65–16·64). However, tourism was also impacted by seasonal variation: ‘[When it is raining] you can’t be able to put your handcraft materials for sale because they will get wet. Tourists … just go back to their homes, yet you were counting on buying food with the money you would have got from them.’

Despite engagement in tourism to generate household income, we found no quantitative evidence of reduced FI among households reporting tourism related-activities, and this was supported by both FGD and key informants. Both the FGD and key informants noted that while tourism activities generate immediate cash income, this did not necessarily translate into improved short- or long-term FI since income is allocated not solely to food and frequently not for lasting food supplies. An FGD participant stated that ‘… most of us run to bars when we get a lot of money.’ A key informant from the Batwa Development Programme highlighted this tendency in communities that participate in tourism: ‘When it’s high season … there are many tourists who come this way; when they [Batwa] are given money … [they] buy food and the rest they drink.’ The quantitative data indicated that alcohol usage was prevalent in the communities; more than 50 % of adults reported drinking regularly. Spending cash income on alcohol was described in most FGD as a substantial contributor to FI. For some families, reducing alcohol consumption during the highly food-insecure season was a coping strategy; ‘if I find a situation getting worse, I stop drinking’ (FGD). However, others mentioned the difficulty of reducing alcohol intake regardless of the severity of FI. A key informant from Bwindi Community Hospital reflected that ‘[a]lcohol is a very big problem … [even] in the general population [non-Batwa] there is a problem of alcohol … we have started the alcohol rehabilitation service at this hospital … [to] tackle that seriously.’

## Discussion

The current study aimed to advance the understanding of the impact of seasonal variation on Batwa food systems and FI. Our findings indicated that although FI was chronic at baseline, there was evidence of seasonal variation producing a magnified famine season. Children were found to be protected from the seasonal impact on FI, with the burden of seasonally magnified FI borne predominantly by adults in the household. Notably, the seasonal experience of FI was not homogeneous, differing in magnitude based on household livelihood strategy and engagement in subsistence agriculture. These findings are consistent with the body of research on the impact of seasonal variation on subsistence and Indigenous populations globally and within Uganda; that is, social determinants of health mediate seasonal impacts^(^ ^14^ ^,^ ^18^ ^,^ ^40^ ^,^ ^47^ ^)^. These results have implications for our ability to generalize trends in climate change vulnerability even within small areas and across seemingly homogeneous sub-populations.

The FGD and key informants consistently reported increased severity of FI during the dry season. The role of seasonal variation and climate on agricultural cycles, specifically harvest timing, yields and quality, is well documented^(^ ^17^ ^,^ ^48^ ^,^ ^49^ ^)^. While the impact of seasonal variation was incremental, even short or minor periods of increased severity of FI can have lasting impacts, particularly on nutrition^(^ ^13^ ^)^. FI exacerbates poor health and increases sensitivity and vulnerability to environmental, social and economic stressors. Increased climatic stressors on the food system will compound the already very high burden of ill health and vulnerable food system among the Batwa^(^ ^18^ ^,^ ^19^ ^)^.

Indigenous populations in Africa and globally consistently have higher rates of negative health outcomes than their non-Indigenous counterparts^(^ ^50^ ^–^ ^53^ ^)^. Land dispossession, lack of compensation and a forced agrarian lifestyle transition underpin much of the vulnerability documented here and have not been sufficiently offset by improvements in socio-economic status and agricultural development to prevent severe inequities in Batwa health compared with the regional average. This narrative of Indigenous loss of land, inequality and struggles adapting to new environments and livelihoods is not unique to the Batwa. Similarly other Indigenous groups that have been evicted from their traditional lands and lifestyles have been documented globally as facing substantial barriers to adopting new livelihoods, with implications for both physical and mental health^(^ ^51^ ^,^ ^54^ ^–^ ^56^ ^)^. Similar to other Indigenous groups who have been relocated or forced into new livelihood options, the Batwa do not have the experience, knowledge, networks or resources that are essential for a successful transition. This is further exacerbated by extreme poverty and continued racial discrimination. While notable improvements have been made since their eviction in 1991, steep social gradients in health persist.

Our qualitative results revealed that the seasonal signal on FI could be partially mediated by participation in tourism, or other forms of cash income labour, in addition to, or as an alternative to, growing crops. Agricultural labour, the most readily available form of employment, is insufficient to enable saving or cash accumulation^(^ ^57^ ^)^. Given these findings, improving knowledge or land access may not be sufficient to improve FI. Access to opportunities for employment in tourism was heterogeneous, as communities closest to the Bwindi Impenetrable National Park were most able to take advantage of this. Tourism, while providing higher income potential, is volatile and climate dependent^(^ ^58^ ^,^ ^59^ ^)^. Key informants reported concern for households choosing not to participate in cultivation; relying on alternative livelihood strategies increased vulnerability, especially if households spent the money immediately. Short-term survival needs, immediate spending and lack of saving prevented reductions in FI from being actualized among Batwa communities. The Batwa, similar to other populations dealing with land dispossession and high poverty, have a high burden of alcoholism^(^ ^60^ ^–^ ^62^ ^)^. Interventions to improve cash revenue opportunities to reduce FI may be unsuccessful unless mainstreamed with consideration of the determinants, prevalence and implications of alcoholism within Batwa communities^(^ ^63^ ^)^. Long-term solutions may be found lacking until poverty, discrimination, and lack of access to land and traditional areas are addressed. In the meantime, a systematic review by Masset *et al*. found that agricultural interventions were successful in increasing food production and consumption; however, there was limited evidence that these interventions increased the nutrition of children under 5 years of age^(^ ^64^ ^)^. As a result, both agricultural extension programming and school feeding programmes are recommended to reduce household FI and improve child nutrition in Batwa communities.

There were a few limitations in the present study the authors would like to address. First, measuring food security is difficult and the measure used here was based on self-reporting and perceptions. Some studies have found that the level of FI may be overemphasized to receive potential aid. However, extensive pilot work was conducted in an effort to integrate communities into the research process and prevent this. Second, the survey administration was conducted during the mid-point of both the dry and wet seasons. While FI was worst during the dry season, the measures of food security taken during the wet seasons did not completely correspond with the harvesting seasons. The harvest season is dependent on the climatic cycles and can fall at varying times dependent on the year. The Batwa may have higher food security than captured herein during their peak harvest time. Additional analysis of seasonal variation was limited due to lack of environmental and weather data. Third, positionality of the researchers may have impacted answers. Every effort was made to negotiate this positionality and reduce the asymmetry between the researchers and participants. However, given the inequality this community faces, power imbalance may have impacted responses by community members.

## Conclusion

While suitable adaptation strategies for vulnerable populations to cope with climatic impacts and increasing their resilience have been established as a priority, it has been distinguished from development, and many funding agencies have specified that funding is to be used for adaptations that specifically address climatic impacts^(^ ^65^ ^,^ ^66^ ^)^. Critics of this approach have highlighted the fact that current poor health and poverty are the largest contributors to sensitivity to climate impacts^(^ ^67^ ^–^ ^69^ ^)^. Given the results presented here, the Batwa are highly vulnerable and sensitive to current seasonal variation. Increased variability predicted with climate change will further increase FI among the Batwa. Multi-scale interventions would be needed in all Batwa communities to reduce current FI and poverty. Reducing Batwa FI would not only reduce the prevalence of undernutrition but would benefit and improve overall health^(^ ^67^ ^)^. The complexity and heterogeneity of seasonal impacts found here support the use of mixed methods in place-based research. While quantitative analysis is useful to identify population-level dynamics and significant effects, qualitative research methods can provide insight into causal mechanisms and explanations for outliers. Further research examining the impact of seasonal variation on agricultural cycles and nutritional outcomes would provide valuable insight for reducing vulnerabilities and implementing appropriate targeted interventions. Additionally, a comparative study between the Batwa and the Bakiga (non-Indigenous) could identify inequities, supporting local efforts to get more health-care access, funding and recognition from the Ugandan Government of Indigenous rights. While the Batwa do have extremely high burdens of illness and poverty, they have an extensive history of adaptation and resilience. Moving forward, the Batwa will need external support to negate historical injustices and partnerships to strengthen their capacity.

## Supplementary material

For supplementary material accompanying this paper visit https://doi.org/10.1017/S1368980016002494. click here to view supplementary material
