# Supplementary material for: Seasonal variation of food security among the Batwa of Kanungu, Uganda
Source: Public Health Nutr. 2016 Sep 13;20(1):1–11. doi: 10.1017/S1368980016002494 (PMC5197730; doi:10.1017/S1368980016002494)
Supplement: Supplementary file 1 [file S1368980016002494sup001.pdf]

Cambridge Journals Purchase order for setting

31 August 2016

Please refer to the proofing instructions for this journal
Proofs MUST be delivered by the time agreed in the service level agreement

article no.	**PHN-RES-2016-0124**	setters no.	1600249	phn	0		running order
first named author	Patterson	author email:	kaitlin.patterson@mail.mcgill.ca				
corresp au	Patterson						
title	Seasonal variation of food security among the Batwa of Kanungu District, Uganda						
doi	10.1017/S1368980016002494	no. of book revs		open_access_article	yes		
submission dates	received 1/2/16	revised 15/7/16	accepted 29/7/16				
article type	**Research Article**						
print colour	no	web colour	yes	Figure 2, Figure 3			
copyright line							
notes for setter							
supplementary materials	Yes	***Supplemental Table 1 and Supplemental Table 2***					
funding_body	CIHR/NSERC/SSHRC and IDRC Tri-Council Initiative on	author country	CA	assoc ed	**MT**		
